# The molecular fidelity of Aβ pathology in 5xFAD and App^NL−F^Psen1^P117L^ mice revealed by cryo-EM

**DOI:** 10.1186/s13024-026-00924-6

**Published:** 2026-01-10

**Authors:** Meinai Song, Hanrun Zheng, Jianting Han, Kaiyu Xu, Deng-Feng Zhang, Qin Cao

**Affiliations:** 1 Bio-X Institutes, Key Laboratory for the Genetics of Developmental and Neuropsychiatric Disorders, Ministry of Education, Shanghai Jiao Tong University, Shanghai, 200030 China; 2 State Key Laboratory of Genetic Evolution and Animal Models, Yunnan Key Laboratory of Animal Models and Human Disease Mechanisms, Kunming Institute of Zoology, Chinese Academy of Sciences, Kunming, Yunnan, 650204 China

## Abstract

Animal models, particularly mouse models, are indispensable for understanding the pathogenic mechanisms and developing therapeutic and diagnostic drugs for Alzheimer’s diseases (AD). While most AD mouse models aim to mimic amyloid-β (Aβ) pathology in their brains, previous research has demonstrated that not all models succeed in reproducing Aβ fibril structures observed in the brains of AD patients. In this study, we determined cryo-electron microscopy (cryo-EM) structures of Aβ fibrils extracted from the brains of two AD models, 5xFAD and App^NL−F^Psen1^P117L^. The former represents one of the most widely used transgenic AD models, while the latter is the third-generation knock-in model designed to address the issues arising from *App* overexpression in conventional models. Our results revealed that fibrils from the 5xFAD mouse exhibit a structure nearly identical to human Type II Aβ fibrils found in AD patients, whereas the App^NL−F^Psen1^P117L^ mouse predominantly possesses Type II Aβ fibrils but also contains another Aβ fibril species. A subtle discrepancy in main chain arrangement at the position of Val36 was noted among all reported human and mouse Type II structures, potentially originating from variations in the brain microenvironment between human and mice. In conclusion, our findings validated the replication of Aβ pathology in 5xFAD and App^NL−F^Psen1^P117L^ mice and provide additional options for selecting appropriate animal models in studies necessitating molecular-level replication of Aβ structures.

**Supplementary Information:**

The online version contains supplementary material available at 10.1186/s13024-026-00924-6.

## Introduction

Alzheimer’s disease (AD) is the most common cause of dementia [1, 2]. The pathological manifestations of AD include extracellular deposition of β-amyloid (Aβ) in the form of diffuse and neuritic plaques, as well as neurofibrillary tangles formed by aggregated hyperphosphorylated tau protein [2, 3]. Under the action of γ-secretase, Aβ peptides with lengths of 38-, 40-, and 42-amino acids are released from the amyloid-β precursor protein (APP) [4]. These Aβ peptides readily aggregate into oligomers and fibrils with a β-sheet conformation [5]. In particular, Aβ42 exhibits higher aggregability and neurotoxicity due to the increased hydrophobicity of its extended C-terminus [6]. To date, three types of Aβ42 fibrils have been identified in human brains. Type I and type II filaments were predominantly found in the brains of individuals with sporadic Alzheimer’s disease (SAD) and familial Alzheimer’s disease (FAD), respectively [7]. Recently type III filaments were observed in the sarkosyl-soluble fraction of an AD patient [8]. Aggregated Aβ has been proven to be a promising target for the development of both therapeutic drugs and diagnostic tracers of AD [9–11].

Animal models are indispensable for both advancing understanding of AD pathogenesis and conducting preclinical evaluation of novel therapeutics. Transgenic mice are the most commonly used AD models, developed mainly through inducing mutations in the Aβ production pathway [12, 13]. While most of these mouse models exhibit Aβ depositions in their brains, previous studies have revealed that not all of them contain Aβ fibrils adopting the same structures with those found in human patients’ brains [7, 14, 15]. Specifically, among the eight mouse models studied so far, only four models, namely App^NL−F^, APP23, ARTE10, and tg-APP_Swe_, have been demonstrated to possess human Type II Aβ fibrils, and only one model, tg-APP_ArcSwe_, has been demonstrated to possess fibrils that are similar to human Type I Aβ fibrils [7, 14, 15]. These findings align with observations that certain AD candidate drugs demonstrated efficacy in preclinical trials using animal models but faced an extremely high failure rate in clinical trials [16, 17]. Therefore, it is important to investigate the structures of Aβ fibrils in the brains of mouse models, particularly the most commonly used ones, to validate the suitability of these models for AD studies focusing on Aβ aggregations.

The 5xFAD mouse model is one of the most commonly used AD models, accounting for 10% of all AD studies with mouse models [13]. These mice express five mutations associated with FAD, including the Swedish (K670N/M671L), London (V717I) and Beyreuther/Iberian (I716V) mutations in APP, as well as the M146L and L286V mutations in presenilin 1 (PSEN1) [18]. Aβ pathology manifests in 5xFAD mice as early as 2 months old, with progressive cognitive deficits evident by 4–5 months [18]. Despite the widespread use of the 5xFAD model in AD research, the structure of Aβ fibrils in the brains of these mice remains unknown.

In addition, alongside these so-called first-generation mouse models based on overexpressing mutant APP or APP/PSEN1 cDNAs, a new strategy involving *APP* knock-in has emerged to address limitations seen in the first-generation models, largely stemming from artifacts related to overproduction of APP fragments [12]. The App^NL−F^ and APP^NL−G−F^ models were the first implementations of this strategy, carrying the Swedish (KM670/671NL) and Beyreuther/Iberian (I716F) mutations, with or without the Arctic (E693G) mutation [19]. While both models exhibit typical Aβ pathology and memory impairments, they each have their respective limitations. The former accurately replicates human Type II Aβ fibrils but develops Aβ pathology slowly (around 18 months), whereas the latter shows rapid Aβ pathology progression but possesses Aβ fibrils with structures differing from those in human patients’ brains, likely due to the Arctic mutation [14, 19]. To overcome the limitations of both models, a third-generation AD mouse model, App^NL−F^Psen1^P117L^, was created, combining APP knock-in with Psen1 knock-in to exhibit accelerated Aβ pathology without the Arctic mutation [20]. However, whether this new model accurately replicates the pathological structure of Aβ fibrils remains uncertain.

In this study, we extracted fibrils from the brains of 5xFAD and App^NL−F^Psen1^P117L^ mice and determined their cryo-EM structures at resolutions of 3.5 Å and 3.2 Å, respectively. Fibrils from both models adopt the human Type II Aβ fibril fold, while fibrils from the App^NL−F^Psen1^P117L^ mouse also contain a minor species that adopts a Type II-like structure. These findings provide essential information for selecting appropriate mouse models for research concerning Aβ pathology and preclinical assessments of Aβ-targeting drugs.

## Results

### Fibril extraction and structure determination

**Fig. 1:**
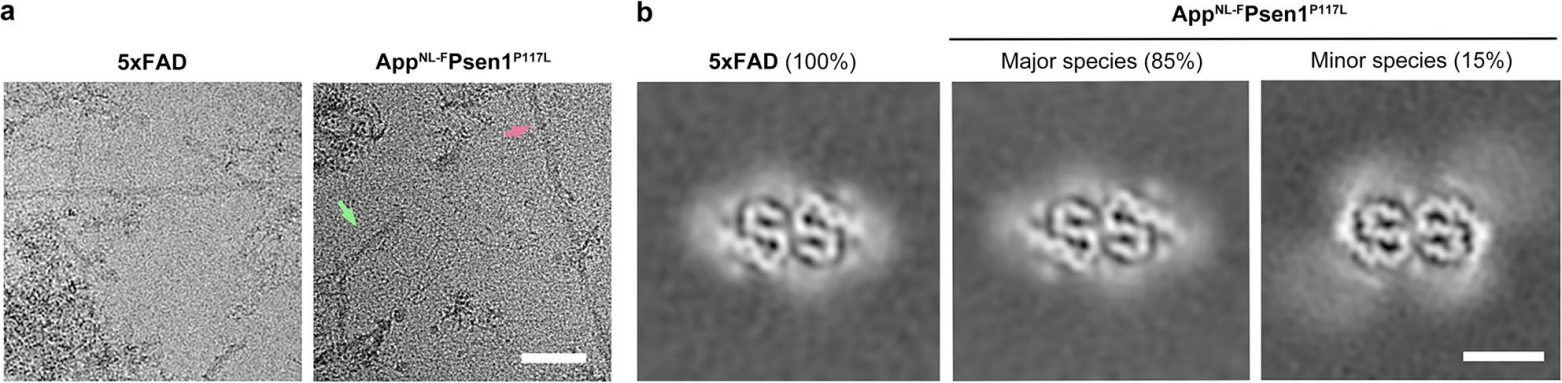
Cryo-EM micrographs and reconstructions. **a**, Representative cryo-EM micrographs of fibrils from 5xFAD and App^NL−F^Psen1^P117L^ mice. Fibrils belonging to the major species and the minor species were indicated with a pale green and pale violet red arrow, respectively. Scale bar = 50 nm. **b**, Central slices of 3D reconstructions of Aβ fibrils from 5xFAD and App^NL−F^Psen1^P117L^ mice (low-pass filtered to a resolution of 5 Å). Scale bar = 5 nm

**Fig. 2:**
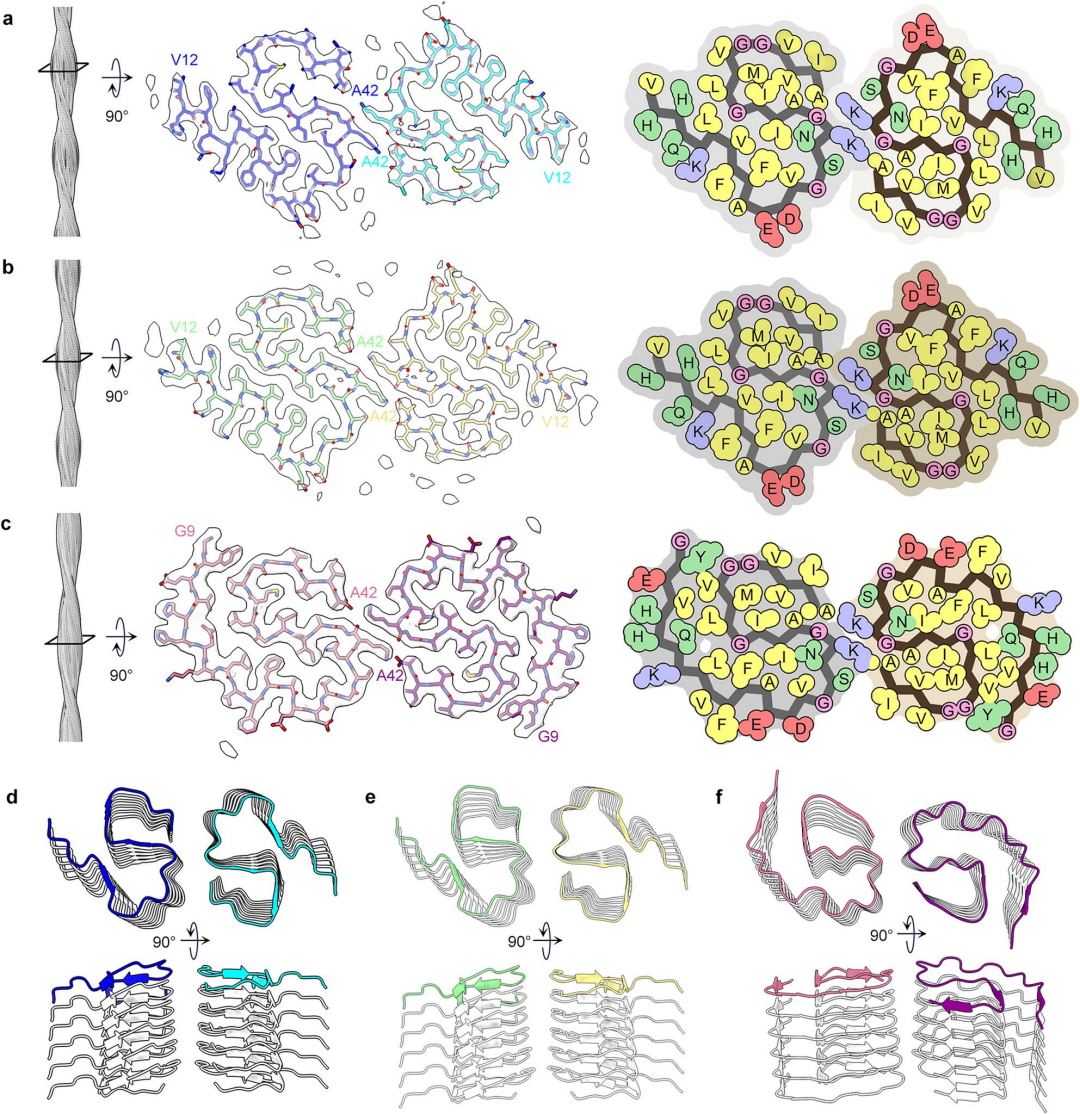
Cryo-EM structure of fibrils from 5xFAD and App^NL−F^Psen1^P117L^ mice. **a-c**, The side view of the fibril reconstruction (left), the cross-sectional layer of the cryo-EM density map with overlaid atomic model (middle), and the space-filling model of the fibril core (right) of fibrils extracted from the brains of 5xFAD (**a**) and App^NL−F^Psen1^P117L^ mice (**b**, major fibril species; **c**, minor fibril species). Residues are color-coded in the space-filling model: hydrophobic (yellow), hydrophilic (green), negatively charged (red), and positively charged (blue). **d-e**, Cartoon representation of the fibril structure with six stacked layers of fibrils from 5xFAD (**d**) and App^NL−F^Psen1^P117L^ (**e**&**f**) mice. The top layer is colored as the schematic in a, b and c

The sarkosyl extraction method [15] were separately employed to perform fibril extractions on 5xFAD and App^NL−F^Psen1^P117L^ mice, and fibrils were found in insoluble fractions (Fig. 1a). Cryo-EM datasets were collected for both models, and two-dimensional (2D) classification revealed that there is only one recognizable fibril species present in the 5xFAD dataset (Fig. S1-S3). In the 2D classes of the App^NL−F^Psen1^P117L^ dataset we observed two fibril species, a major species exhibiting morphology similar to that of the 5xFAD species (accounting for 85% of the total recognizable fibrils) and a minor species (accounting for the remaining 15%, Fig. S1). We determined the 3D structure of these fibril species at a resolution of 3.5 Å, 3.2 Å, and 3.4 Å, respectively (Fig. 2a-c, Fig. S4). The structure determination and model building statistics are listed in Table 1. The data processing workflow is illustrated in Fig. S2&S3.

**Table 1:** Cryo-EM data collection, refinement and validation statistics of mice Aβ fibrils

	5xFAD (EMD-65825, PDB 9WAO)	App^NL−F^Psen1^P117L^ Major species (EMD-65826, PDB 9WAP)	App^NL−F^Psen1^P117L^ Minor species (EMD-67622, PDB 21FB)
**Data collection and processing**			
Magnification	×130,000	×130,000	×130,000
Voltage (kV)	300	300	300
Electron exposure (e^–^/Å^2^)	40	40	40
Defocus range (µm)	1.5–2.5	1.5–2.5	1.5–2.5
Pixel size (Å)	0.932	0.932	0.932
Symmetry imposed	C_2_	C_2_	C_1_
Helical rise (Å)	4.77	4.76	2.37
Helical twist (°)	-3.2	-3.2	178.9
Initial particle images (no.)	426,271	1,608,927	1,410,854
Final particle images (no.)	25,555	35,234	16,317
Map resolution (Å)	3.5	3.2	3.4
FSC threshold	0.143	0.143	0.143
Map resolution range (Å)	200 − 3.5	200 − 3.2	200 − 3.4
**Refinement**			
Initial model used (PDB code)	7Q4M	9WAO (5xFAD)	9WAO (5xFAD)
Model resolution (Å)	3.7	3.4	3.5
FSC threshold	0.5	0.5	0.5
Model resolution range (Å)	200 − 3.7	200 − 2.4	200 − 3.5
Map sharpening *B* factor (Å^2^)	107	95	98
Model composition			
Nonhydrogen atoms	2,712	2,712	3,012
Protein residues	372	372	408
Ligands	-	-	0
*B* factors (Å^2^)			
Protein	39.46	59.35	80.06
Ligand	-	-	-
R.m.s. deviations			
Bond lengths (Å)	0.011	0.006	0.007
Bond angles (°)	1.096	0.643	0.800
**Validation**			
MolProbity score	2.53	1.76	1.92
Clashscore	25.36	10.51	5.63
Poor rotamers (%)	0	0	0
Ramachandran plot			
Favored (%)	86.21	96.55	87.50
Allowed (%)	13.79	3.45	12.50
Disallowed (%)	0	0	0

### Cryo-EM structure of the Type II Aβ fibrils from both mouse models

We found that the structures of the fibrils from the 5xFAD mouse and the major species from the App^NL−F^Psen1^P117L^ mouse closely resemble that of the Type II Aβ filaments extracted from Alzheimer’s disease patient brain tissue [7] (Fig. 1b). Specifically, the fibrils from both mouse models consist of two protofilaments, with each layer comprising two symmetrically related polypeptide chains. Each chain exhibits an S-shape conformation and consists of Val12-Ala42 of Aβ (Fig. S5). The S-shape conformation is stabilized by two hydrophobic clusters composed of residues Leu17, Val18, Phe20, Val24, Ile31, and Leu34, as well as residues Ala30, Ile32, Met35, Val40, and Ala42 (Fig. 2a&b). The twofold symmetry axes are centered between Lys28 sidechains, which form inter-subunit salt bridges with the Ala42 carboxylate groups. The interlayer stacking of the fibrils is stabilized by the arrangement of three β-strands into three parallel β-sheets (Fig. 2d&e).

### Cryo-EM structure of the minor fibril species from the App^NL−F^Psen1^P117L^ mouse

**Fig. 3:**
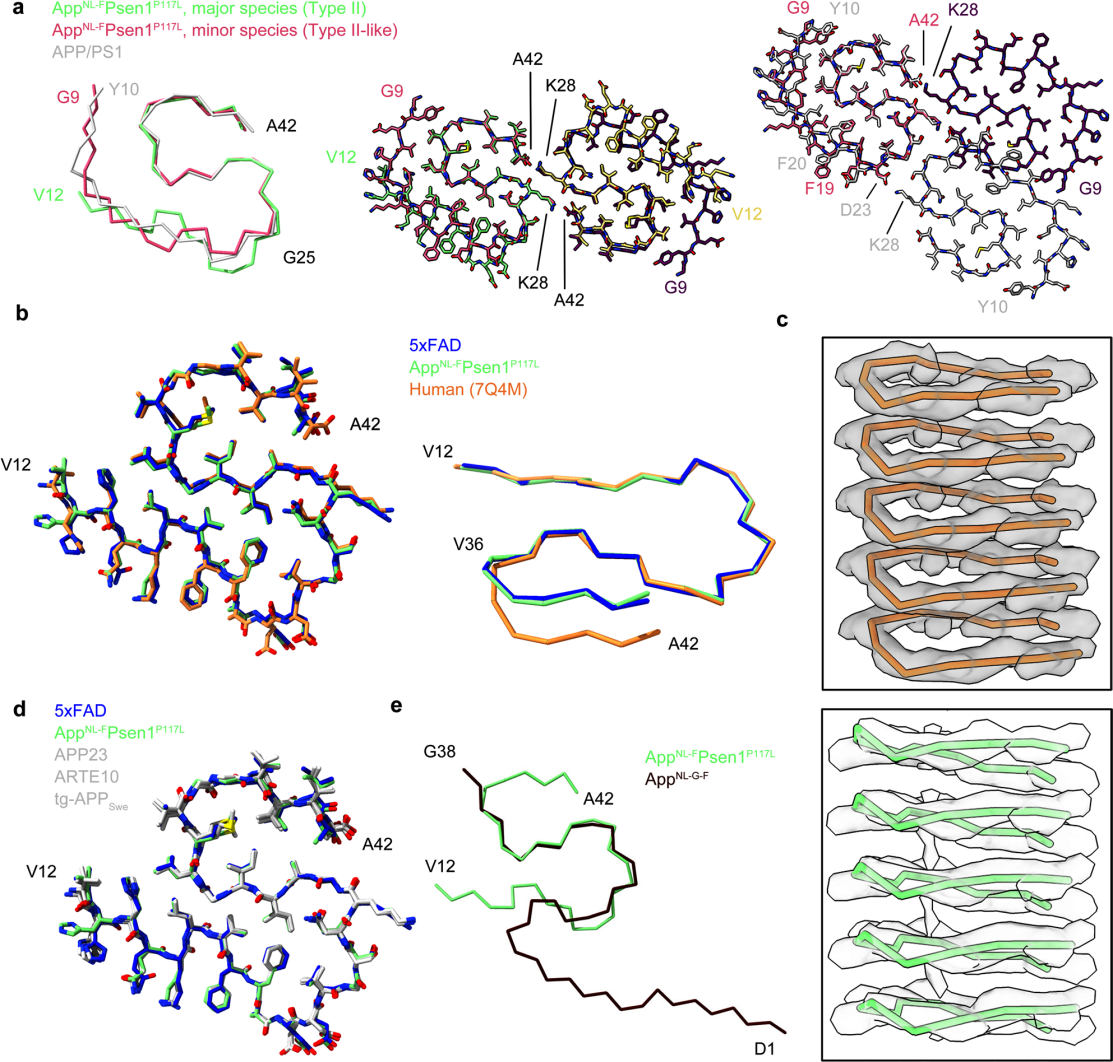
Comparison of Aβ fibrils derived from human and different mouse models. **a**, Comparison of the major and minor fibril species from the App^NL−F^Psen1^P117L^ mouse and fibrils from APP/PS1 mice (PDB 8OL3). **b**, Comparison of the Type II Aβ fibrils extracted from 5xFAD, App^NL−F^Psen1^P117L^ and human (PDB 7Q4M) brains. **c**, Map-model overlay of the Ala30-Ala42 segments of Type II Aβ fibrils from human (top) and App^NL−F^Psen1^P117L^ mice (bottom). **d**, Comparison of the Type II Aβ fibrils extracted from 5xFAD, App^NL−F^Psen1^P117L^, APP23 (PDB 8OL2), ARTE10 (PDB 8OL5), and tg-APP_Swe_ (PDB 8OL6) mice. We note that the structure of Aβ fibrils from App^NL−F^ mouse is not available on the PDB, thus it is not included in this comparison. **e**, Comparison of the Aβ fibrils extracted from App^NL−F^Psen1^P117L^ and App^NL−G−F^ (PDB 8BFA) mice

The minor fibril species from the App^NL−F^Psen1^P117L^ mouse are composed of Gly9-Ala42 of Aβ, with the region spanning Gly25-Ala42 closely resembling the type II filaments, including the dimer interfaces (Figs. 2c, f and 3a). The overall fold of each protofilament is similar to the murine type III fibrils observed in APP/PS1 and ARTE10 mice [15], but with differences in the orientation of Phe19 and Phe20 as well as the dimer interfaces (Fig. 3a). These observations suggest that this fibril species can be defined as Type II-like fibrils and may represent an intermediate state between murine type III and type II Aβ fibrils, potentially linked to the unique microenvironment in the brain of the App^NL−F^Psen1^P117L^ mouse. While this species adopts a structure that distinct from that of the pathological Aβ fibrils in humans, we believe the App^NL−F^Psen1^P117L^ mouse largely, though not fully, replicates the Aβ pathology in AD, with Type II Aβ fibrils as the major fibril species and the minor species also replicating the core region of Type II Aβ fibrils.

In summary, through cryo-EM structure determination, we found that fibrils extracted from the brains of 5xFAD and App^NL−F^Psen1^P117L^ mice are predominantly Type II Aβ fibrils, with a minor fibril species present in the App^NL−F^Psen1^P117L^ mouse.

### Structural comparison with human Type II Aβ fibrils

Structural comparisons between the fibrils extracted in this study and those obtained from human brain revealed that, when viewed parallel to the fibril axis, the fibrils from the 5xFAD mouse and the major fibril species from the App^NL−F^Psen1^P117L^ mouse exhibit an almost identical structure to human Type II Aβ fibrils [7] (Fig. 3b). However, when observed perpendicular to the fibril axis, we found a difference in the main chain direction at the position of Val36 between human and mice fibrils, resulting in a shift of approximately 4 Å of residues 37–42 when aligning residues 12–34. (Fig. 3b, Table S1). This variation affects the inter-subunit salt-bridge formation between Lys28 and Ala42. Specifically, in human Type II Aβ fibrils, Lys28 from layer i interacts with the carboxyl groups of Ala42 from both layer i and i-1; whereas in 5xFAD and App^NL−F^Psen1^P117L^ Aβ fibrils, Lys28 from layer i interacts with the carboxyl groups of Ala42 from layer i-1 and i-2 (Fig. S6a, Table S2).

To further investigate that whether the variation in main chain directions observed here is a common feature distinguishing human and mice fibrils, we compared the structures of all reported Type II Aβ fibrils from both human and mouse brains [7, 8, 14, 15, 21, 22]. The results suggest that all human Type II Aβ fibrils, including the protofilament that adopt Type II fold observed in the human Type III Aβ fibrils, exhibit the same main chain direction, while all mouse Type II Aβ fibrils also exhibit the same main chain direction (Fig. S6b, Table S1-2). The main chain arrangements in these fibril structures were further validated by the cryo-EM maps, showing a good alignment between the main chain directions and the corresponding maps (Fig. 3c and Fig. S6c). This alignment suggests that the variations in main chain directions are not due to misinterpretation of the cryo-EM map. Meanwhile, the observation that all maps are well separated between layers suggests that there is no random distribution of heterogenous main chain arrangements in each fibril species. Taken together, while Type II fibrils from humans and mice are largely identical, we observed a clear clustering in main chain directions between human and mice fibrils, which may be attributed to subtle variations in the environments in human and mice brains.

### Structural comparison with other mouse Aβ fibrils

To date, four mouse models have been reported to exhibit Type II Aβ fibrils, namely App^NL−F^ (the atomic model has not been deposited to PDB) [7], APP23 (PDB ID 8OL2), ARTE10 (PDB ID 8OL5), and tg-APP_Swe_ (PDB ID 8OL6) [15]. Structural comparisons with these fibrils indicate that 5xFAD and App^NL−F^Psen1^P117L^ mouse possess the same Type II Aβ fibrils as these four models (Fig. 3d). Notably, the App^NL−F^Psen1^P117L^ mouse model, developed subsequent to the App^NL−F^ and App^NL−G−F^ models, maintains the Type II configuration observed in App^NL−F^ but differs from App^NL−G−F^ (Fig. 3e). The structural variance in App^NL−G−F^ fibrils may be attributed to the Arctic mutation (E22G), as the backbone conformation differs at the position of residue 22 between App^NL−G−F^ and other two APP knock-in models (Fig. 3e).

### Additional densities in 5xFAD and App^NL−F^Psen1^P117L^ Type II fibrils

Previous research has demonstrated that human Aβ42 fibrils exhibit additional densities that could potentially indicate ligand binding. Notably, densities near Glu22 and Asp23 are of particular significance, as ligand binding at these sites might mitigate the electrostatic repulsions of the negatively charged residues [7]. Consistent with these earlier observations, Type II fibrils obtained from 5xFAD and App^NL−F^Psen1^P117L^ mice also display additional densities, including those adjacent to Glu22 and Asp23 (Fig. 1b, Fig. S6d). These findings suggest that these ligands may play a crucial role in the formation of pathological Aβ fibrils, and absence of these ligands could be a primary reason for the inability to replicate the same fibril structures in vitro.

## Discussion

Cerebral deposition of aggregated Aβ stands as a hallmark of AD. While the relationship between Aβ accumulation and AD pathogenesis has sparked debate over the past three decades, recent research, notably the success of two Aβ-targeting drugs in mitigating cognitive decline in AD patients [9, 10], reinforces the pivotal role of Aβ in both the mechanistic and therapeutic studies of AD. Numerous animal models, particularly mouse models, have been engineered to facilitate explorations into disease mechanisms and therapeutic approaches. Most AD mouse models have been tailored to replicate key pathological features of AD, with a specific focus on Aβ pathology. However, owing to the polymorphic nature of amyloid fibrils, Aβ fibrils generated outside the brains of AD patients might not faithfully replicate identical pathological structures. Hence, investigating the fibril structures in the brains of these mouse models is imperative to ensure their fidelity in reproducing Aβ pathology. In this study, we extracted fibrils from the 5xFAD and App^NL−F^Psen1^P117L^ mouse models and determined their structure using cryo-EM. Our results disclose that the former model possesses a single fibril species closely resemble human Type II Aβ fibrils, while the later model contains Type II Aβ fibrils as the major species and Type II-like fibrils as the minor species.

While four mouse models, namely App^NL−F^, APP23, ARTE10, and tg-APP_Swe_, have been reported to predominantly harbor Type II Aβ fibrils in their brains [7, 15], the inclusion of the 5xFAD and App^NL−F^Psen1^P117L^ models in this category remains highly significant. Firstly, 5xFAD mice stand out as one of the most commonly used AD models. Their advantages include commercial availability, the ability to mimic numerous AD symptoms at an early age, and extensive research conducted on its pathology and behavior. For instance, in terms of the Aβ accumulation timeline, Aβ deposition starts at 18, 6, and 10–12 months of age in App^NL−F^, APP23, and tg-APP_Swe_ mice, respectively [20, 23, 24], whereas in 5xFAD mice, Aβ deposition starts as early as 2 months [18]. Therefore, opting for the 5xFAD model can significantly reduce time needed for Aβ related studies with animal models. Secondly, App knock-in mice are deemed superior models as they replicate AD pathology without the excessive production of APP. Among these, the App^NL−F^Psen1^P117L^ model emerges as the most optimal App knock-in model available, capable of rapidly accumulating Aβ without the Arctic mutation. Thus, our study has validated the molecular fidelity of Aβ aggregation in these two models, offering additional choices for selecting mouse models for the future AD research that necessitates precise replication of Aβ structure, such as preclinical studies of Aβ-targeting drugs or tracers.

Our structural analysis has unveiled a discrepancy in main chain arrangements between human and mouse Type II Aβ fibrils (Fig. 3b & Fig. S6). This discrepancy emerges as a crucial feature that distinguishes human and mouse fibrils. We note that this discrepancy was cross validated with fibrils from five different mouse models and five human cases of AD (Fig. S6b, Table S1-3). This cross-model consistency suggests that the observed main chain arrangements are a common feature in mice under diverse sexual, genetic, and phenotypic background. Further studies are required to investigate the key factors underlying this discrepancy and to ascertain whether it implies that murine models cannot fully replicate the pathogenesis of AD. It is worth noting that, despite this discrepancy, we maintain that mouse Type II fibrils serve as a valid replication of human Type II fibrils for drug and tracer development. Our structure superimposition has demonstrated that residues 37–42 of layer i-1 in mouse fibrils closely align with those of layer i in human fibrils (Fig. S6e). Thus, the identities and positions of side chains on the fibril surfaces of both human and mouse fibrils are nearly identical, except for a small region near Gly37, which is unlikely to significantly impact the specificity of ligand binding.

Given that both the 5xFAD and App^NL−F^Psen1^P117L^ models contain Type II Aβ fibrils, we are currently lacking a method to reproduce Type I Aβ fibrils accurately. The tg-APP_ArcSwe_ model has been demonstrated to only partially emulate Type I fibrils [15]. It is understandable that mouse models tend to replicate Type II fibrils more readily, as most models involve mutations associated with FAD. Further studies are required to establish reliable models for SAD, potentially by replicating the brain microenvironment of SAD patients instead of introducing hereditary mutations.

It is worth noting that the limitation of this study, similar as the previous studies on other AD model mice [7, 15], is that all these structures were determined from fibrils of aged mice, long after the onset of Aβ deposits in that model (Table S3). This strategy may ensure that these determined structures represent the end-stage conformation of Aβ aggregation, but information about the conformation of Aβ at the early stage of aggregation is missing. Further studies are required to extract fibrils from mice of different ages and compare the structure of these fibrils. These studies may facilitate our understanding of the dynamic aspects of Aβ accumulation in the brain.

In summary, our structural analysis reveals that Aβ42 fibrils derived from both 5xFAD and App^NL−F^Psen1^P117L^ mouse brains exhibit high similarity to human Type II fibrils. This congruence provides a structural rationale for utilizing these models in developing Alzheimer’s disease therapeutics and amyloid tracers, expanding available preclinical options and strengthening translational relevance.

## Methods

### 5xFAD and App^NL−F^Psen1^P117L^ mice

Brain from one female 10-month-old heterozygous 5xFAD mouse on a C57BL/6J background were used for fibril extraction and cryo-EM structure determination. 5xFAD mouse express five mutations associated with FAD, including the Swedish (K670N/M671L), London (V717I) and Beyreuther/Iberian (I716V) APP mutations and the M146L and L286V mutations in PSEN1. Beginning at 2 months of age, these mice form abundant extracellular deposits [18].

The App^NL−F^Psen1^P117L^ knock-in mouse model (C57BL/6-App < tm2(NL-F)Tcs > Psenl < cml (P117L)Tcs>, No.RBRC11518), provided by the RIKEN BRC through the National BioResource Project of the MEXT, Japan, was generated by crossing App^NL−F^ mice with Psen1^P117L^ mice. These mice are homozygous for both the App and Psen1 mutations, with Aβ deposition starts as early as 2 months of age [20]. Brain from one male 14-month-old App^NL−F^Psen1^P117L^ mice was used for fibril extraction and cryo-EM structure determination.

### Fibril extraction

Fibrils were extracted from 5xFAD and App^NL−F^Psen1^P117L^ mice according to a previously reported protocol [15] with minor adjustments. In brief, approximately 400 mg of brain tissue was homogenized using a glass tissue grinder with 20 volumes (w/v) of extraction buffer consisting of 10 mM Tris-HCl, pH 7.5, 800 mM NaCl, 10% sucrose, 1 mM EGTA. Subsequently, the homogenate was supplemented with 2% sarkosyl and 0.25 mg/ml each of DNase/RNase, followed by 1 h incubation at 37 °C with gentle agitation. After a 10 min centrifugation at 10,000 ×g, the supernatants were collected and further centrifuged at 100,000 ×g for 60 min. The pellets were resuspended in extraction buffer (1 ml/g of initial tissue weight) and subjected to centrifugation at 3,000 ×g for 5 min. The resultant supernatants underwent threefold dilution in 50 mM Tris-HCl, pH 7.5, 0.15 M NaCl, 10% (w/v) sucrose, and 0.2% (w/v) sarkosyl, followed by ultracentrifugation at 100,000 ×g for 30 min. The pellets were resuspended in 20 mM Tris-HCl, pH 7.4, and 50 mM NaCl for further negative staining and cryo-EM analysis.

### Cryo-EM data collection and processing

3 µl of fibril samples were applied to holey carbon film (Quantifoil 1.2/1.3, 200 mesh) and incubated for 2 minutes. The grids were blotted with filter paper for 5.5 s and plunge-frozen in liquid ethane using a Vitrobot Mark IV (Thermo Fisher Scientific). Data was collected on a FEI Titan Krios G3i transmission electron microscope (Thermo Fisher Scientific) operated at 300 kV with a Falcon 4i detector in counting mode. The total accumulated dose was 40 e^−^/Å^2^ per image, and the nominal physical pixel size was 0.932 Å/pixel. A total of 2,373 and 7,726 micrographs were collected for the fibrils from 5xFAD and App^NL−F^Psen1^P117L^ mice, respectively.

Data processing of the fibrils followed the workflow outlined in Fig. S2&S3. Motion correction and contrast transfer function (CTF) estimation were performed using MotionCor2 [25] and CTFFIND-4.1.827 [26], respectively. Helical reconstruction was carried out using RELION 4.0 [27]. Particles were automatically picked with Topaz v0.2.5 [28] and extracted at box sizes of 360 and 720 pixels. The inter-particle distance was maintained at 36 pixels.

Two-dimensional (2D) classification of particles with both 360-pixel and 720-pixel boxes was performed separately, revealing a single fibril morphology in the 5xFAD dataset and two fibril morphologies in the App^NL−F^Psen1^P117L^ dataset. In the case of the 5xFAD dataset, the initial 3D reconstruction was conducted with the 360-pixel particles derived from the most optimal 2D classes. A featureless cylinder served as the initial reference for the 3D reconstruction, and the helical parameter was calculated as follows: the cross-over distance was measured to be 262 Å from the 2D classes of 720-pixel boxes (Fig. S1); the point group symmetry was set to C_2_ based on the apparent mirror symmetry of the 2D classes; the initial helical rise was estimated as 4.8 Å based on the common feature of amyloid fibrils; the initial helical twist was calculated as 180° × helical rise ÷ cross-over distance, resulting in a value of 3.3°; the helical twist was set to -3.3° because we assume these fibrils are left-handed, as it is more common for amyloid fibrils. We acknowledge that we cannot confirm the handedness of the fibrils at the current stage, and the selection of handedness should not affect the monomer structure of the fibrils. The first round of 3D classification (k = 3) was performed, and particles from the best 3D class were selected for an additional round of 3D reconstruction (k = 1) with either C_2_ or 2-start helical symmetry. Inspection of the resulting maps suggested that C_2_ symmetry yielded better separation of layers, leading to the utilization of C_2_ symmetry for further reconstructions. The helical parameters were refinement for the C_2_ symmetry. The search range for helical twist was set from − 3.6° to -3.0° with the search step of 0.01°, whereas the search range for helical rise was set from 4.7 Å to 4.9 Å with the search step of 0.05 Å. The refined helical twist was − 3.2°, and the refined helical rise was 4.77 Å. Subsequently, the second round of 3D classification was performed, utilizing particles selected from the first round of 3D classification, refined helical parameters, and map from the best 3D class of the previous round as a reference. Particles from the best 3D class of the second round were used for the golden-standard 3D refinement, generating a cryo-EM map with a resolution of 3.6 Å. CTF refinement and Bayesian polishing were conducted, followed by a final round of golden-standard 3D refinement to produce a final map with a resolution of 3.5 Å.

In the case of the major fibril species of the App^NL−F^Psen1^P117L^ dataset, the data processing adhered to a parallel methodology as described above with the following modifications. First, two rounds of 2D classification were performed instead of one. Second, helical parameter refinement was carried out after rather than between the two rounds of 3D classifications (Fig. S3). For the minor fibril species of the App^NL−F^Psen1^P117L^ dataset, the data processing also followed the same protocol with the following modifications. First, particles were selected based on the 2D classification of the 720-pixel box-size particles. These particles were re-extracted with a 360-pixel box and used for the initial 3D reconstruction. Second, during the reconstruction with either C_2_ or 2-start helical symmetry, the 2-start helical symmetry resulted in better layer separation. Hence, 2-start helical symmetry was used for further reconstruction. The resolution of the final maps for the major and minor fibril species of App^NL−F^Psen1^P117L^ dataset was 3.2 Å and 3.4 Å, respectively. The resolutions for both datasets were estimated using the 0.143 Fourier shell correlation (FSC) resolution cutoff.

### Atomic model Building

The initial model of fibrils from 5xFAD mice was generated by docking a single chain of the human Type II Aβ fibril structure (PDB 7Q4M) as a rigid body. This initial model was then manually modified using COOT [29]. The resulting model unambiguously accommodates residues 12–42 of Aβ within the cryo-EM map, validating the identity of these fibrils as Aβ. The model was expanded to include 6 layers and refined using phenix.real_space_refine [30]. The final model was validated using MolProbity [31]. For the fibrils from App^NL−F^Psen1^P117L^ mice, a single chain from the fibril structure of 5xFAD mice was docked to generate the initial model, and the following procedure adhered to the same aforementioned protocol.

## Supplementary Information

Below is the link to the electronic supplementary material.

Supplementary Material 1

## Data Availability

Cryo-EM maps and atomic models of fibrils from 5xFAD and App ^NL-F^ Psen1 ^P117L^ mice present in this study have been deposited into the Worldwide Protein Data Bank (wwPDB) and the Electron Microscopy Data Band (EMDB) with accession codes PDB 9WAO, EMD-65825 (fibrils from 5xFAD), PDB 9WAP, EMD-65826 (major fibril species from App^NL-F^ Psen1^P117L^), and PDB 21FB, EMD-67622 (minor fibril species from App^NL-F^ Psen1^P117L^ ), respectively. Any other relevant data are available from the corresponding author upon reasonable request.
