# Supplementary material for: The molecular fidelity of Aβ pathology in 5xFAD and AppNL−FPsen1P117L mice revealed by cryo-EM
Source: Mol Neurodegener. 2026 Jan 10;21:10. doi: 10.1186/s13024-026-00924-6 (PMC12882323; doi:10.1186/s13024-026-00924-6)
Supplement: Supplementary file 1 — Supplementary Material 1 [file 13024_2026_924_MOESM1_ESM.docx]

**Title: The molecular fidelity of Aβ pathology in 5xFAD and App^NL-F^Psen1^P117L^ mice revealed by cryo-EM**

**Authors:** Meinai Song^1^, Hanrun Zheng^1^, Jianting Han^1^, Kaiyu Xu^2^, Deng-Feng Zhang^2*^, Qin Cao^1*^

**Affiliations:**

^1^Bio-X Institutes, Key Laboratory for the Genetics of Developmental and Neuropsychiatric Disorders, Ministry of Education, Shanghai Jiao Tong University, Shanghai, 200030, China

^2^State Key Laboratory of Genetic Evolution and Animal Models, Yunnan Key Laboratory of Animal Models and Human Disease Mechanisms, Kunming Institute of Zoology, Chinese Academy of Sciences, Kunming, Yunnan, 650204, China

^*^Correspondence to: Qin Cao, email: [caoqin@sjtu.edu.cn](mailto:caoqin@sjtu.edu.cn); Deng-Feng Zhang, email: [zhangdengfeng@mail.kiz.ac.cn](mailto:zhangdengfeng@mail.kiz.ac.cn)


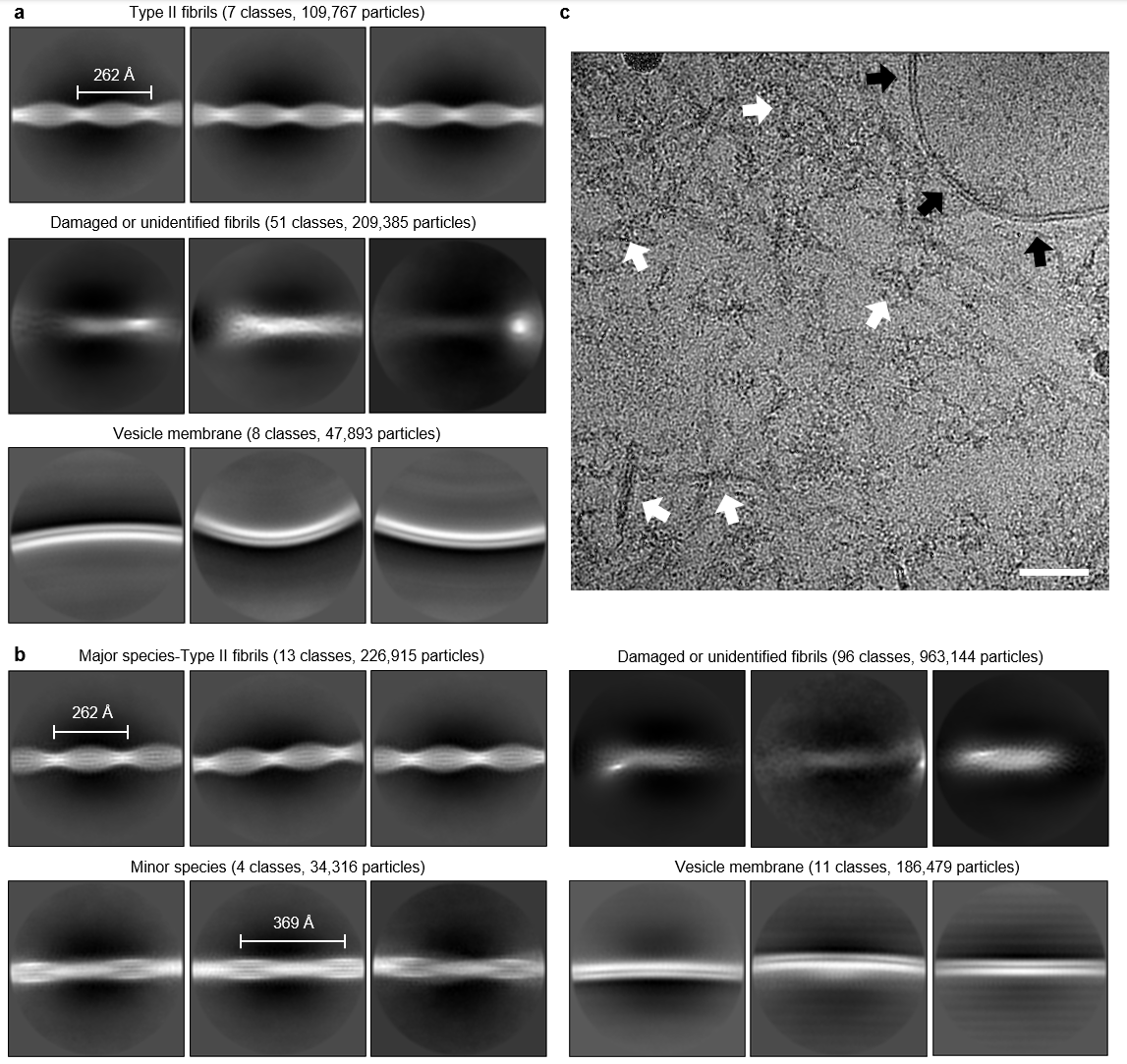


**Figure S1 Particle picking and two-dimensional (2D) classification during data processing. a&b,** Representative 2D classes from the 5xFAD (a) and App^NL-F^Psen1^P117L^ (b) datasets, with the total numbers of 2D classes and particles belonging to these classes labeled. Particles used in 2D classification were extracted with a 720-piexel box. The cross-over distances of twisted fibrils are measured and labeled. **c**, Representative cryo-EM micrographs from the App^NL-F^Psen1^P117L^ dataset. Particles belonging to 2D classes representing damaged or unidentified fibrils (white arrows) or vesicle membrane (black arrows) are indicated. Scale bar = 50 nm.


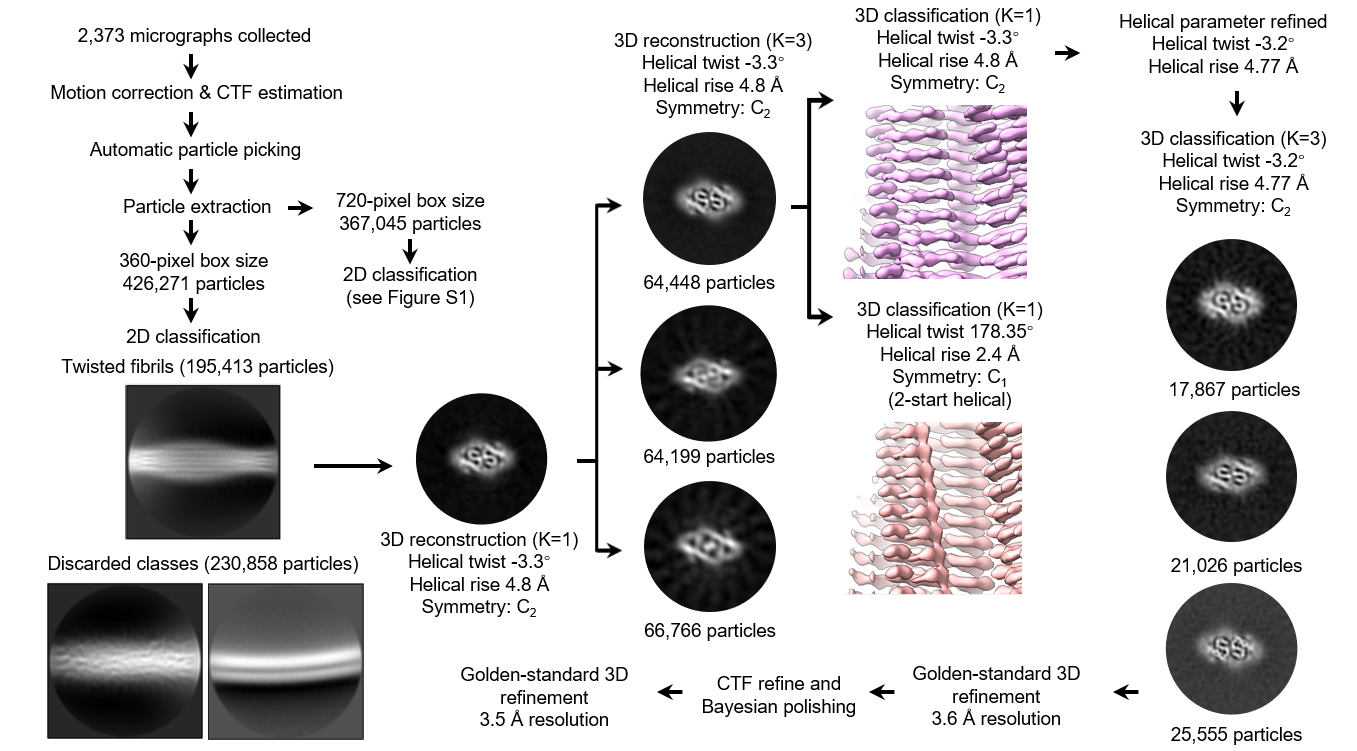


**Figure S2 Cryo-EM data processing workflow for Aβ fibrils from 5xFAD mice.**


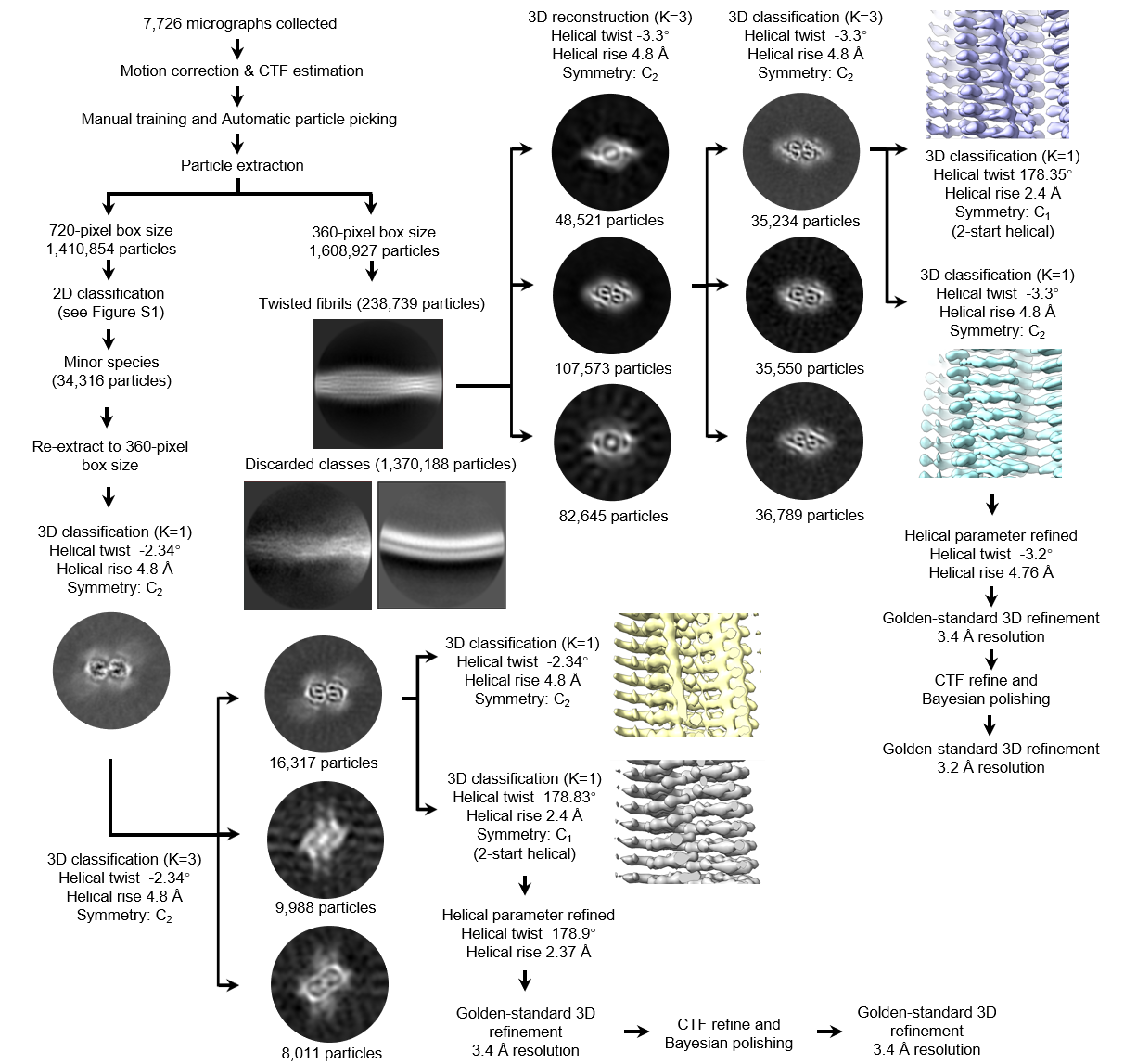


**Figure S3 Cryo-EM data processing workflow for Aβ fibrils from App^NL-F^Psen1^P117L^ mice.**


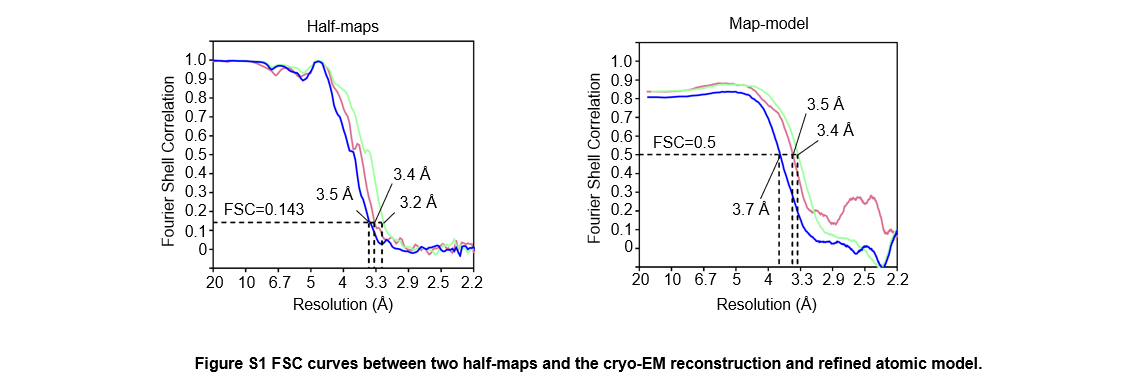


**Figure S4 FSC curves between two half-maps and the cryo-EM reconstruction and refined atomic model.** Curves are colored in blue (5xFAD), pale green (App^NL-F^Psen1^P117L^, major species), and pale violet red (App^NL-F^Psen1^P117L^, minor species).


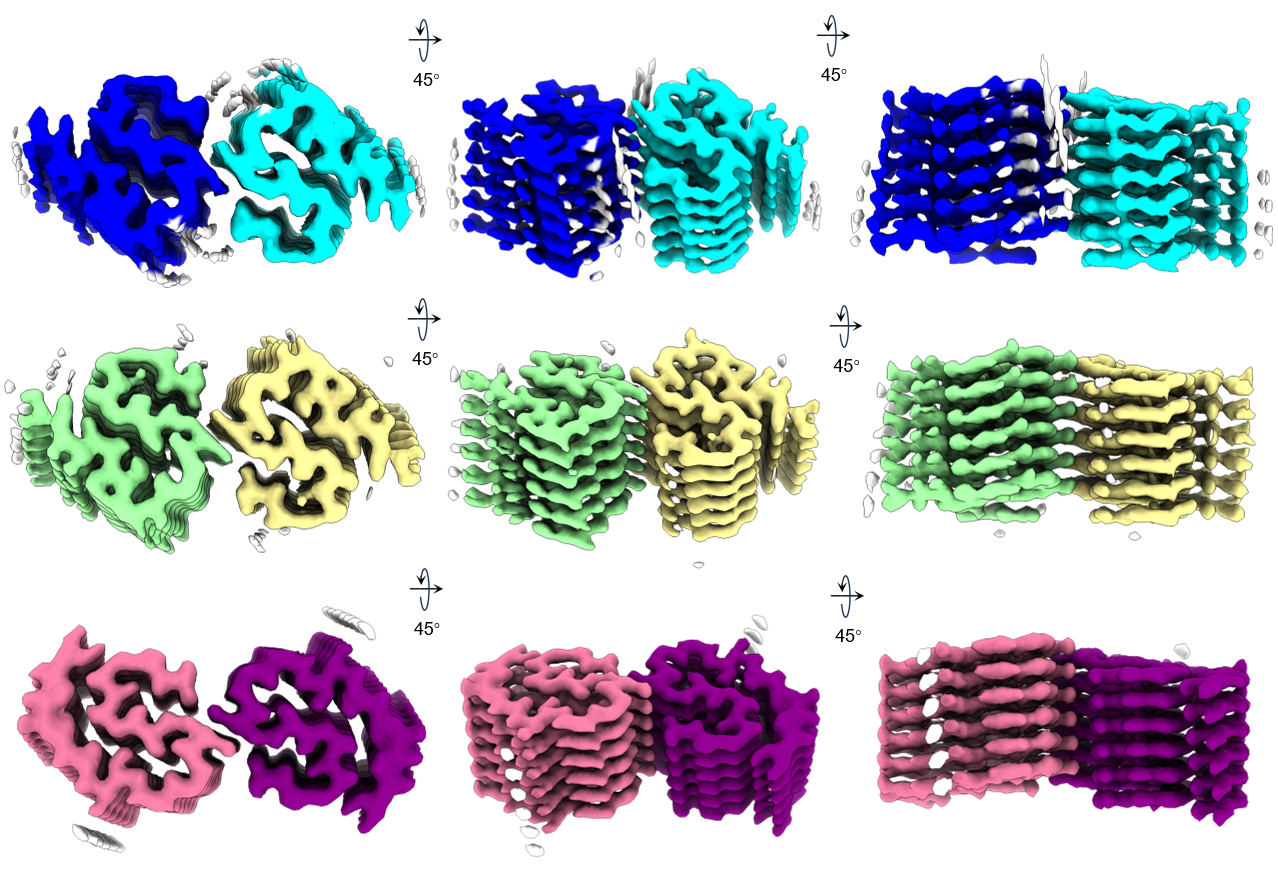


**Figure S5 Cryo-EM map of mouse Type II fibrils.** Different views of the cryo-EM maps obtained from 5xFAD and App^NL-F^Psen1^P117L^ mice with six layers shown. The maps of the two protofilaments are colored in blue and cyan (5xFAD), pale green and khaki (App^NL-F^Psen1^P117L^, major species), or in pale violet red and purple (App^NL-F^Psen1^P117L^, minor species), respectively. Unmodeled additional densities in both protofilaments are colored in white.


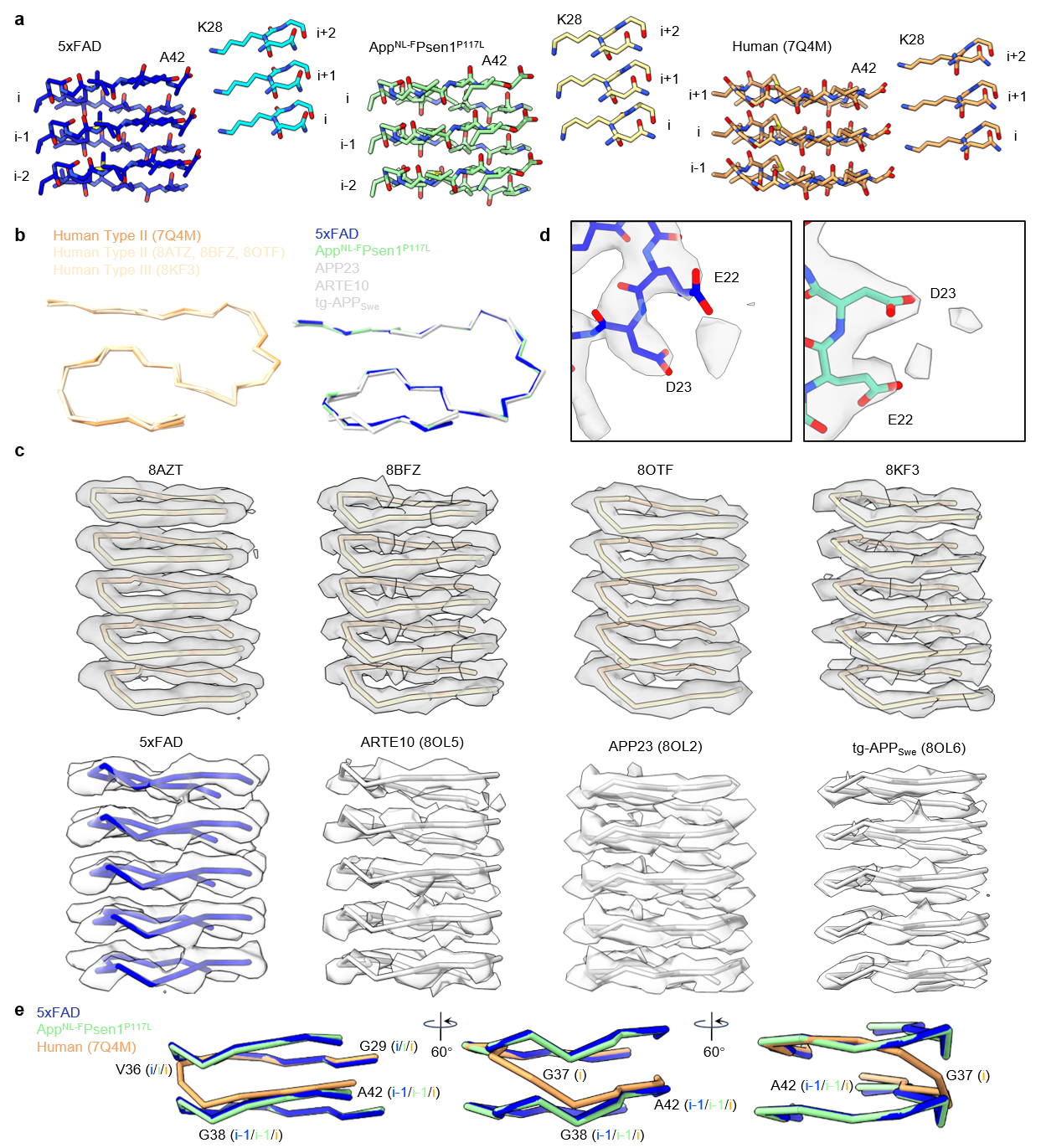


**Figure S6 Additional analysis of Type II fibrils. a**, Inter-subunit salt-bridges between Lsy28 and Ala42 from 5xFAD, App^NL-F^Psen1^P117L^ mice and human (7Q4M). Three stacked layers are shown in sticks and colored according to the schematic in Fig. 2&3. **b**, Comparisons of main chain arrangements of human (left) and mouse (right) Type II fibrils. **c**, Side views of map-model overlay of Ala30-Ala42 segments of Aβ fibrils with Type II fold from the brains of human and mice. **d**, Additional densities near Glu22 and Asp23 in fibrils from 5xFAD (left) and App^NL-F^Psen1^P117L^ (right) mice. **e**, Comparison of residues 29-42 of layer i/i-1 in mouse fibrils and layer i in human fibrils.

**Table S1 Distances (in Å) between Cα atoms of indicated residues from different Type II Aβ fibrils.**

|  |  | Val36 | Gly37 | Gly38 | Val39 | Val40 | Ile41 | Ala42 |
| --- | --- | --- | --- | --- | --- | --- | --- | --- |
| Mouse model (PDB ID) | 5xFAD (9WAO)* | - | - | - | - | - | - | - |
|  | App^NL-F^Psen1^P117L^ (9WAP) | 0.18 | 0.20 | 0.20 | 0.44 | 0.20 | 0.33 | 0.72 |
|  | ARTE10 (8OL5) | 0.23 | 0.25 | 0.56 | 0.43 | 0.47 | 0.76 | 1.40 |
|  | APP23 (8OL2) | 0.45 | 1.89 | 1.91 | 1.25 | 0.58 | 0.70 | 1.42 |
|  | tg-APP_swe_ (8OL6) | 0.73 | 1.99 | 2.04 | 1.47 | 0.53 | 0.86 | 1.63 |
| Human | PDB 7Q4M | 0.75 | 3.56 | 3.73 | 4.33 | 4.50 | 4.60 | 3.78 |
|  | PDB 8AZT | 0.89 | 3.27 | 3.31 | 3.94 | 4.13 | 4.40 | 3.60 |
|  | PDB 8BFZ | 0.51 | 3.44 | 3.82 | 4.35 | 4.53 | 4.56 | 4.37 |
|  | PDB 8OTF | 0.91 | 3.34 | 3.46 | 4.11 | 4.25 | 4.39 | 3.54 |
|  | PDB 8KF3 | 0.56 | 3.28 | 3.53 | 4.34 | 4.60 | 4.96 | 4.09 |

*All structures below were aligned with this structure; residues 12-34 were aligned.

**Table S2 Distances (in Å) between Lys28 of layer i and Ala42 of indicated layers in different Type II Aβ fibrils.**

|  |  | i-3 | i-2 | i-1 | i | i+1 |
| --- | --- | --- | --- | --- | --- | --- |
| Mouse model (PDB ID) | 5xFAD (9WAO) | 7.44 | **3.52*** | **3.60** | 7.40 | 11.90 |
|  | App^NL-F^Psen1^P117L^ (9WAP) | 7.26 | **3.77** | **3.70** | 7.65 | 12.17 |
|  | ARTE10 (8OL5) | 6.82 | **3.27** | **2.45** | 6.90 | 11.62 |
|  | APP23 (8OL2) | 7.83 | **3.52** | **2.28** | 6.62 | 11.29 |
|  | tg-APP_swe_ (8OL6) | 8.09 | **3.52** | **2.26** | 6.61 | 11.29 |
| Human | PDB 7Q4M | 12.46 | 7.55 | **3.16** | **3.20** | 7.27 |
|  | PDB 8AZT | 13.36 | 8.87 | **5.01** | **3.86** | 6.79 |
|  | PDB 8BFZ | 12.16 | 7.52 | **3.40** | **3.17** | 6.30 |
|  | PDB 8OTF | 12.20 | 7.52 | **3.20** | **3.14** | 7.46 |

*Two of the layers with the shortest distance in each model were labeled in bold, indicating the formation of the salt bridge.

**Table S3 Information of the mice used in determining the Type II Aβ fibrils**

| **Mouse models** | **Sex** | **Month of Age** | **Onset of Aβ deposit (months)** |
| --- | --- | --- | --- |
| 5xFAD | female | 10 | 2 |
| App^NL-F^Psen1^P117L^ | male | 14 | 2 |
| ARTE10 | female | 24 | 3 |
| APP23 | male | 21 | 6 |
| tg-APP_swe_ | male | 22 | 10-12 |
